# Effectiveness of Cognitive Behavioral Therapy on Spiritual Well-Being and Emotional Intelligence of the Elderly Mourners

**Published:** 2017-04

**Authors:** Abas Solaimani Khashab, Hosain Ghamari Kivi, Davod Fathi

**Affiliations:** 1 Department of Education and Psychology, Allameh Tabatabaei University, Tehran, Iran.; 2 Department of Education and Psychology, Mohaghegh Ardabili University, Ardabil, Iran.; 3 Department of Education and Psychology, Rehabilitation Counseling, Mohagheghardabili University, Ardabil, Iran.

**Keywords:** *Cognitive Behavioral Therapy*, *Elderly Bereavement*, *Emotional Intelligence*, *Spiritual Well-Being*

## Abstract

**Objective: **Grief is one of the most painful experiences of the humans after linking emotions. In the literature of trauma, grief and mourning can be seen on many topics. Intervention and treatment of grief seems necessary as the period of mourning is prolonged. Thus, this study aimed at understanding the effectiveness of cognitive behavioral therapy on spiritual well-being and emotional intelligence in the elderly bereavement.

**Method:** This was an experimental study with pre-and posttest design, and control group. The population of this study was the elderly mourners in city of Ardabil in 15-2014. After conducting clinical interviews and diagnostic tests using the sampling method, 30 elderly mourners selected. Spiritual Well-Being questionnaire and Emotional Intelligence questionnaire were used for data collection. The questionnaire and pretest-posttest were used in this study. Data were analyzed using multivariate analysis of covariance.

**Results:** The results of the data analysis revealed that cognitive behavioral therapy increased spiritual well-being and emotional intelligence of the mourners was not significantly different between the 2 groups (P<0.01). However, the means of Spiritual Well-Being and Emotional Intelligence at pretest was not significant in the intervention group compared with the control group (P>0.05).

**Conclusion:** Method of cognitive behavioral therapy helps confront the emotional drain and grief acceptance, increasing the spiritual well-being and emotional intelligence of the elderly bereavement.‏

Grief is a natural reaction that most people experience (1). In this state, severe internal anguish and distress occur in response to the loss of a person or a particular opinion or thought. Grief can be both internal (thoughts and feelings) and external (bereavement-related behaviors, such as crying) (2). Worden categorized the symptoms of grief into 5 groups as follow (2009): physical, cognitive, emotional, and spiritual (3). To be resolved, these symptoms require treatment.

In accordance with the model of coping with bereavement (that paying due attention to the emotional and spiritual symptoms can have significant impact on the healing process), in the present study the concept of spiritual well-being was selected to represent the spiritual symptoms. In the recent years, spirituality has been considered an important aspect of humanistic approach associated with well-being and the healing process. Spiritual well-being is one of the aspects of human health that provides coordinated and integrated relationship between the internal forces; and it is characterized by attributes such as stability in life, peace, balance and harmony, and feeling a close relationship with self, God, the society, and the environment (4). “Spiritual dimension” has been incorporated into the 10th revision of the International Statistical Classification of Diseases and Related Health Problems (ICD-10) published by World Health Organization, which means that just like the physical, psychological and social dimensions, which are interrelated and affect each other, spiritual dimension is also associated with other dimensions of health (5). 

Along with physical, mental and social dimensions, spiritual health is now considered as one of the aspects of human health, and it not only improves the general health but also coordinates other dimensions of well-being, and thus enhances psychological adaptability and function (6). Spiritual well-being is defined as human’s spiritual experience throught one of the two following perspectives: religious well-being, and existential well-being (7). In a study by Livneh, Lott, and Antonak (2004), it was found that spirituality plays an important role in coping with the stress caused by a disease (8). In a study by Hamid Keikhosravi, Babamiri, and Dehaghani (2012), the relationship between mental health and spiritual intelligence with the resiliency of students was assessed and it was concluded that providing supportive factors (such as religion and spirituality) to enhance the mental health and spiritual intelligence can lead to increased resilience (9). In addition to spirituality, the ability to regulate emotions can also be effective in individual adaptability and resiliency.

Moreover, according to the dual process model of coping with the loss, representing the emotional symptoms has been selected as the concept of emotional intelligence. Mayer and Salovey (1997) stated that emotional intelligence involves the ability to recognize and respond appropriately to emotions and feelings of others as well as to stimulate awareness, regulate, and control their emotional responses (10). Goleman (1995) making some changes to the definition of Mayer and Salovey, introduced emotional intelligence as another aspect of intelligence, which includes the awareness of feeling and its use for making appropriate decisions in life, bearing psychological trauma, and inhibition of mental disorders. (11). One study showed that emotional intelligence is a predictor of life satisfaction in people (12). Teroler (2003) in his study on reaction to the death of a family member and using humor found that families who used humor showed emotional comfort and reduced discomfort in dealing with the death of their family members. Reducing the symptoms of grief and improving emotional and spiritual health in bereaved people is very important. The signs of mourning (3) in addition to the over whelming grief, have caused different cultures to find ways to help their members to leave behind the grief and adapt to life after the death of a dear person. Social networks and cultural context supports are helpful in reducing the stress and discomforts of the bereaved person to adapt with changes after the loss of a family member and return to life. Accordingly, applying other therapies to balance the bereaved persons’ life and their adaptation to new conditions is of prime importance. Cognitive-behavioral therapy (CBT) is one of these therapies.

Cognitive-behavioral therapy approach is one of the most popular treatments that many researches are devoted to it. In this approach, the clients are supported in developing skills to change behavior, relations with others, problem solving, discovering distorted thoughts and ideas, challenging the body and changing unhelpful beliefs and attitudes, and cognitive restructuring (13). The nature of cognitive-behavioral approach is that its cognitive products are intermediaries between situations and emotional, behavioral, and physiological responses, so this approach is a significant expansion of stimulus and response patterns of human behavior (14). A research to evaluate the effectiveness of cognitive behavioral therapy on adaptation of students with abnormal grief on a sample of 28 people concluded that cognitive-behavioral therapy has a positive impact on the adjustment of the students to grief (15). The emphasis on spirituality in the treatment is one of the effective factors that can influence the improvement of social protection and adaptation in matters relating to health (16). One study showed that cognitive behavioral counseling has a positive effect on spirituality and mental health in the patients (17). According to some findings, spirituality can ameliorate the disease and increase survival, and it seems those who rely on spirituality to deal with injuries and trauma have better reactions and responses to the treatment (18). On the other hand, the dual model of coping with the loss emphasizes the emotions and emotional consequences in reducing the symptoms of grief. Fathi et al. (2014) in their study on the effectiveness of Ellis rational-emotional cognitive therapy on emotional intelligence found that cognitive therapy could improve emotional intelligence among the staff of Tehran Municipality (19). Research on the effectiveness of group cognitive therapy on emotional intelligence revealed that this treatment could improve emotional intelligence (20). In a study to assess the effects of training the emotional intelligence components on mental health, it was found that training the components of emotional intelligence could improve the mental health of people (21).

In general, the sadness of mourning experience has devastating impact on mental, spiritual and emotional health of the individuals. In Iran, little research has been conducted on the treatment of bereaved people. Thus, the present study examined the effectiveness of cognitive- behavioral therapy on emotional intelligence and spirituality of the mourners.

## Materials and Methods

The participants in this study were 30 mournful old adults who were selected from the mournful elderly living in the nursing homes of Ardebil (Iran) in 2014 and 2015. The clinical interview and available sampling method were used. They were assigned into one of two groups: 15 in the experimental group and 15 in the control group. Thirty elderly people available on an alphabetic order were categorized into two groups. The participants aged 60 to 75 years old. in the first session, the spiritual well-being and emotional intelligence pretest questionnaire was administered for both groups. The experimental group was exposed to an independent variable. At the end, the spiritual well-being and emotional scales was assessed as the posttest. The study criteria included experiencing a loss and grief, being an elderly, and lack of psychiatric and non-psychiatric drugs. The exclusion criteria were as follow: not willing to participate in the study, and disability to do the research project. In addition, before the implementation of the treatment, the aim of the study was discussed with the participants. 

***Instrument***

Spiritual Well-Being Questionnaire (SWB): This questionnaire, which has been developed by Paulotzin & Baffer (2009), consists of 20 questions and measures two dimensions. Each of the questions has a Likert scale of 1 to 6 ranging from totally disagree to totally agree; 10 items measure religious well-being, and the other 10, measure the existential well-being. Each of the religious and existential scores ranges from 10 to 60; however, there is no ranking for these 2 subgroups and judgments is based on the obtained scores. The sum of these scores is the score of spiritual well-being, which ranges from 20 to 120 and includes terms such as “I feel that life is a positive experience” and “I have a special meaningful relationship with God”. Paulotzin & Baffer (2009) has reported a Cronbach's alpha of 0.89 for the whole scale, 0.87 for the religious domain and 0.78 for the existential domain. In addition, in a study on cancer patients, validity of this SWB questionnaire has been confirmed through content validation and its Cronbach's reliability has been reported as 0.82 (21).

Emotional Intelligence Questionnaire: Petrides & Farnham designed this questionnaire in 2001 based on blending patterns of emotional intelligence. The basic form of this questionnaire has 114 questions, 30 items, and 51 scales. Each of the questions has a 7- point Likert scale from strongly disagree to strongly agree and assesses 4 scales including optimism, understanding emotions, emotional control, and social skills (23). This questionnaire covers questions such as the following: "I tolerate sadness of others even if they are my relatives", "I am tolerant of other`s anger and disgust", and "I get worried in certain situations, regularly." Petrides & Farnham (2003) have reported that the internal consistency of the questionnaire was 86%. Moreover, the significant positive correlation between this questionnaire and Eysenck Personality Profile questionnaire indicates the acceptable concurrent validity of the questionnaire (24, 25 and 26). Yousefi and Safari (2009) and Alborzi and et al. (2013), using Cronbach's alpha, have reported the reliability of the questionnaire to be from 76% to 88% (25, 26).

## Results

Before the analysis of covariance (ANCOVA), the Levene's test was performed to assess the homogeneity of the variances and verify the compliance of data with ANCOVA assumptions (Table 3). The results revealed no significance for any variable, so the condition of homogeneity of the intergroup variance was satisfied, and it was therefore acceptable to use ANCOVA (Tables 4 & 5).

The results of statistical characterized Lambda Wilkes also showed that group effect on spiritual well-being and emotional intelligence as 2 criteria was significant (Wilks' Lambda = 0.27, F = 36.19, P>0.001). This test also certified the administration of ANCOVA.

Results of ANCOVA revealed that the mean score of spiritual well-being of the experimental group (F = 9.25) was significantly higher than that of the control group. Moreover, and there was a significant difference between the pretest and posttest data (0.001> P). The table below shows the stages of cognitive behavioral therapy for treatment of mourning:

**Table 1:** Structure of Cognitive Behavioral Therapy (CBT) on Mourners

**First Session:** Interviewing the Mournful Elderly, Developing a Safe and Supportive Relationship, Sympathizing, Explaining the Goals, Investigating the Condition and Situation Qualitatively, and Expressing the Emotions
**Second Session:** Psychological Restating for 1 Hour including Normalization of the Responses, Modifying and Catharsis of the Emotions in a Supporting Environment, Expressing Emotions, and Reactions in Exposure Time
**Third Session:** Familiarizing the Participants with the Concept of "Emotion" and "Depression", their Symptoms and Effects on Intensifying the Symptoms of Bereavement, Giving Homework
**Forth Session: **Instructing and Doing Relaxation to Reduce the Symptoms of Emotional Arousal
**Fifth Session:** Using Imagination including Replacing the Intrusive Memorials of the Lost One with the Pleasant Thoughts and Using the Strategy of Minimizing and Removing the Image of Memorials to Repel and Suspend the Intrusive Thoughts
**Sixth Session:** Applying the Techniques of Avoiding the Annoying Events and Memorials including Gradual Desensitization
**Seventh Session:** Exchanging the Roles of the Deceased Person and the Bereaved, Expressing the Emotions and Feelings of the Bereaved Individual
**Eighth Session:** Teaching the Skills and Restoring the Routine Daily Activities and Art Therapy (Imagination and Symbolization of the Lost Person in the Works…) that Cause the Bereaved to Replace Him/Herself Emotionally with the Deceased One

**Table 2:** The Mean and Standard Deviation of Spiritual Well-Being and Emotional Intelligence of the Experimental and the Control Groups in Pretest and Posttest

**Variable**	**Group**	**Pre- Post-Test**		**Posttest**	
		**M**	**SD**	**M**	**SD**
Spiritual well-being	Control group	69.31	2.07	69.76	2.02
	Experimental group	70.23	3.01	77.41	1.84
Emotional intelligence	Control group	*81.73*	*2.3453E1*	*93.46*	*1.6172E1*
	Experimental group	*80.93*	*2.61*	*98.06*	*1.57*

**Table3:** Levene's Test of Homogeneity of Variances, for the Variables of Spiritual Well-Being and Emotional Intelligence in the Experimental and Control Groups

**Df1**	**Df2**	**Α**	**F**
1	28	0/36	1/58
1	28	0/15	2/18

**Table 4:** MANOVA Results for the Main Impact of the Group’s Variant on Dependent Variants

	**Test**	**Value**	**F**	**Significance**
**Group**	Pillai's Trace	0.72	36.19	0.001
	Wilks' Lambda	0.27	36.19	0.001
	Hotelling's Trace	2.68	36.19	0.001
	Roy's Largest Root	2.68	36.19	0.001

**Table5:** Results of ANCOVA on the Mean Score of Spiritual Well-Being and Emotional Intelligence in the Pretest and Posttest for the Experimental and the Control Groups

**Source of Differences**	**Sum of the** **Squares**	**Degree** **of Freedom**	**Mean of the** **Squares**	**F**	**Significance**	**Power of the ** **Test**
**Pretest effect of** **spiritual well-being**	7.22	1	7.22	0.027	0.78	0.05
**Effect of intervention**	2499.10	1	2499.10	9.25	0.00**	0.25
**Pre-test effect of on ** **Emotional intelligence**	2.718	1	2.718	0.058	0.81	0.002
**effect of intervention**	2536.78	1	2536.78	6.45	0.01*	0.19

P<0.001

Table 2 shows the mean and standard deviation of the pre-test and post-test variables in both groups show. Before the analysis of covariance (ANCOVA), the Levene's test was performed to assess the homogeneity of variances and verify the compliance of data with ANCOVA assumptions (Table 3). The results showed no significance for any variable, so the condition of homogeneity of inter-group variance was satisfied, and it was therefore acceptable to use ANCOVA

The result of statistical characterized Lambda Wilkes (Table4) also showed that group effect on Spiritual Well-Being and Emotional Intelligence as two criteria was significant (Lambda Wilkes=0.27, F=36.19, P>0.001). This test also certified the administration of ANCOVA.

Results of ANCOVA in Table5 showed that the mean score of spiritual well-being(F=9.25) and emotional intelligence (F = 6.45) of the experimental group is significantly higher than that of control group, and there is a significant difference between pre-test and post-test data (0.001> P).

## Discussion

This study aimed to determe the effectiveness of group cognitive behavior therapy on mental health and emotional intelligence, and the results showed this treatment was effective.

These results are consistent with the findings of Koszycki, Raab, Aldosary, and Bradwejn (2010) and Barrera, Zeno, Bush, Barber, and Stanley (2012) (27, 28). According to Elmer, McDonald, and Friedman (2003), spiritual intelligence reduces the illness and increases longevity. Moreover, apparently spirituality-oriented people respond better to treatment when dealing with traumas and injuries (29). Also, a study on the relationship of spiritual intelligence and social intelligence with the death anxiety in elderlies has shown that spiritual intelligence reduces the fear of death (12). Another study on the definition and conceptualization of spiritual well-being has reported that measuring spiritual well-being should not be focused on our view of spiritual health, but rather on personal understandings, actions, emotions, and welfare of people (30). This conclusion is in some way consistent with the emphasis of the model of cognitive-behavioral therapy on the changes in cognitive, emotional, and behavioral attributes and the argument that modifying these dimensions can be effective in assessing people's spiritual health. Cognitive behavioral therapy help the patient accept their grief, emphasizes the support factors, and strengthens the cognitions and beliefs of the bereaved person and can alter their view toward the grieving experience and strengthen their spiritual well-being. Given the strong link between bereavement and spiritualties in the Iranian culture (via concepts such as decree and destiny, will of God, etc.), it can be argued that strengthening the process of accepting and coping with bereavement increases people's spiritual well-being.

The findings of this study revealed the positive impact of group cognitive-behavioral therapy on improving emotional intelligence of the bereaved persons. These findings are consistent with those of Cramer & Cupshik (1993), Lewinson & gotlip (1995), Moller, Rabe, & Nortje (2000), Harris & Dryden (2006), and Fathi et al. (2014) (31,32,33,19). Grief causes emotional imbalance in the bereaved person, and after the sudden shock caused by the bereavement, symptoms such as anger, sadness, and frustration appear in the bereaved person. Sometimes, the symptoms can be seen in the bereaved persons for a long time and they cannot cope with the impact of bereavement. In such circumstances, the ability to regulate emotions is essential to counter the impact of grief. Emotions are a key in cognitive-behavioral therapy. Wright, Bescko and Teize (2006) argue that one of the most important signs of flowing with the automatic thoughts in the mind is having strong emotions (34). With respect to the relationship between emotion, cognition, and behavior in the cognitive-behavioral model, identifying automatic thoughts and resolving these thoughts lead to emotion regulation. Moreover, those receiving this kind of treatment are trained to regulate their emotions in different situations. Furthermore, the emphasis of cognitive behavior therapy on cognition, emotion, and behavior in different situations and tasks such as thoughts registration, behaviors and emotions experienced by the person in different situations cause them to be familiarized with emotional functions and be more aware of their emotions. Emotions emergence and emotional discharge on other members of the group and ways of dealing with these emotions by members created a suitable environment to learn the skills to cope with negative emotions and gain different ways and solutions to regulate and control their emotions. In general, group cognitive- behavioral therapy with emphasis on enhancing the capacity of mourners in understanding their feelings and emphasis on motivating them, can control their level of excitement and regulate their relations with others. Thus, group cognitive behavior therapy has a positive impact on the emotional intelligence of bereaved 

persons and improves their emotional intelligence.

## Limitations

The research limitations were the selection method, limited sample size, and the impossibility of controlling some limitations on quasi-experimental designs. Moreover, no research has been conducted in Iran on the effectiveness of group cognitive-behavioral therapy on emotional intelligence and spiritual health of the bereaved elderly. Thus, the results of the present study could not be compared with any similar study in Iran.

## Conclusion

In general, it seems necessary to use the group cognitive-behavioral therapy to improve symptoms of the mourners, to increase their spiritual health and emotional intelligence, and to empower them to cope with their loss and develop more patience against the impact of grief. The findings of this study in addition to fill in the gap of the existing theories in this area can be useful to clinical centers, psychiatric hospitals, clinicians, and researchers. Finally, it is recommended that while replicating this research using other population groups, the role of cognitive-behavioral therapy to treat other symptoms of grief also be examined.
